# Addressing unmet needs in relapsed/refractory multiple myeloma: an Italian Delphi consensus on current challenges and emerging therapies

**DOI:** 10.3389/fonc.2026.1756247

**Published:** 2026-02-13

**Authors:** Michele Cavo, Sara Bringhen, Gabriele Buda, Francesco Di Raimondo, Pellegrino Musto, Massimo Offidani, Maria T. Petrucci, Elena Zamagni, Renato Zambello, Francesco Berruto, Rachele Freddi, Paola La Malfa

**Affiliations:** 1 Department of Medical and Surgical Sciences, University of Bologna, Bologna, Italy; 2 Division of Hematology, Azienda Ospedaliera (AO) Santa Croce e Carle di Cuneo, Cuneo, Italy; 3 Unità Operativa (UO) Hematology, Department of Clinical and Experimental Medicine, University of Pisa, Azienda Ospedaliero-Universitaria Pisana (AOUP), Pisa, Italy; 4 Hematology Division, Azienda Ospedaliero-Universitaria (AOU) “Policlinico-S. Marco”, University of Catania, Catania, Italy; 5 Department of Precision and Regenerative Medicine and Ionian Area, “Aldo Moro” University School of Medicine, Bari, Italy; 6 Unit of Hematology and Stem Cell Transplantation, Azienda Ospedaliero-Universitaria Consorziale (AOUC) Policlinico, Bari, Italy; 7 Hematology Clinic Stem Cell Transplant Unit at Azienda Ospedaliero-Universitaria (AOU) delle Marche, Ancona, Italy; 8 Azienda Ospedaliera-Universitaria Policlinico Umberto I, Roma, Italy; 9 Istituto di Ricovero e Cura a Carattere Scientifico (IRCCS) Azienda Ospedaliero-Universitaria di Bologna, Seràgnoli Institute of Hematology, Bologna, Italy; 10 Department of Medical and Surgical Sciences, Bologna University, Bologna, Italy; 11 Hematology, Padua University School of Medicine, Padua, Italy; 12 Real World Solutions, IQVIA Solutions Italy S.R.L., Milan, Italy

**Keywords:** belantamab mafodotin, ciltacabtagene autoleucel, daratumumab-refractory, Delphi consensus, lenalidomide-refractory, relapsed/refractory multiple myeloma, unmet therapeutic needs

## Abstract

**Background:**

Despite advances in treating multiple myeloma, the management of patients with relapsed or refractory disease remains a major clinical challenge. The increasing use of novel agents in frontline therapy has led to a growing population refractory to key drugs such as lenalidomide and daratumumab, resulting in limited effective options in subsequent lines. Moreover, the increasing prevalence of double-refractory cases highlights a challenge with limited evidence guiding treatment choice. In Italy, the anticipated introduction of innovative immunotherapies offers new hope, yet their real-world impact remains uncertain. This study aimed to establish expert consensus on current challenges, emerging therapies, and address unmet needs in the Italian clinical landscape.

**Methods:**

A modified Delphi process was employed to reach consensus regarding the current and future management of relapsed/refractory multiple myeloma in Italy. One hematology expert served as opinion leader, while a panel of eight experts participated in two rounds of online surveys and a final virtual consensus meeting. Panelists were blinded to others throughout the process. In each survey round, experts provided their level of agreement on statements regarding epidemiology of multiple myeloma in Italy, treatment patterns in clinical practice, and unmet needs in second and subsequent therapy lines. The final meeting allowed discussion and consolidation of responses.

**Results:**

Most statements (28/31, 90%) achieved consensus after two Delphi rounds, while three required further discussion and agreement in the final meeting. Experts reached consensus on the epidemiology of multiple myeloma in Italy and preferred treatment patterns for relapsed/refractory disease. They consistently highlighted the limited efficacy and safety of available second- and third-line therapies for relapsed/refractory multiple myeloma, regardless of prior exposure, recognizing a substantial unmet clinical need due to the lack of satisfactory options, particularly in specific populations including double-refractory patients to lenalidomide and anti-CD38 agents. The anticipated introduction of innovative immunotherapies was identified as a promising opportunity to address these gaps and improve outcomes.

**Conclusion:**

Despite multiple available agents and combinations, effective treatment of all patients with relapsed/refractory multiple myeloma remains a major unmet need. Given their distinct mechanism of action, immunotherapies hold significant potential to address this gap and improve clinical outcomes.

## 1 Introduction

Worldwide, multiple myeloma is the third most frequent hematologic malignancy after non-Hodgkin lymphoma and leukemias (1). In 2022, its incidence in Europe was estimated to 50,092 new cases; there were 31,969 deaths and the estimated 5-year prevalence was 149,397, corresponding to 20 affected individuals per 100,000 (1). In Italy there are no dedicated registries for multiple myeloma and epidemiologic data are often scattered or derived from general databases or registries. A systematic analysis of the Global Burden of Disease study 2017 focused on Italy reported 5,983 incident cases of multiple myeloma and 3,638 deaths in 2017 (2). According to this analysis, the age-standardized incidence rate per 100,000 person-years was 5.4 in males and 3.5 in females; the age-standardized death rates per 100,000 person-years were 2.8 and 1.9, respectively. According to the estimates by the Italian Association of Medical Oncology (AIOM) and the Italian Association for Tumor Registries (AIRTUM), in 2024 approximately 34,000 individuals were affected by multiple myeloma in Italy, while approximately 6,600 new patients were diagnosed with the disease (3).

Treatment options for multiple myeloma have expanded over the past two decades, leading to remarkable improvements in overall survival (4–6). Nevertheless, the natural history of the disease continues to be marked by sequential phases of relapse due to increasing drug resistance over time (7). The three main classes of novel agents that have transformed the therapeutic landscape of multiple myeloma include immunomodulatory agents (thalidomide, lenalidomide, pomalidomide), proteasome inhibitors (bortezomib, carfilzomib, ixazomib), and monoclonal antibodies targeting CD38 (daratumumab and isatuximab) and SLAMF7 (elotuzumab) antigens. These drugs have been combined to form doublet, triplet, and even quadruplet regimens which are currently included into the latest European Hematology Association-European Myeloma Network (EHA-EMN) and National Comprehensive Cancer Network (NCCN) guidelines as standard-of-care therapies for newly diagnosed patients, both candidates (aged less than 70 years and non-frail) and non-candidates for autologous stem cell transplant (ASCT) (8–10). Among recommended upfront treatments, those currently reimbursed in Italy and preferred in most patients include the four-drug regimen daratumumab-bortezomib-thalidomide-dexamethasone followed by lenalidomide maintenance for ASCT candidates, and the three-drug combination daratumumab-lenalidomide-dexamethasone for non-candidates to ASCT (8–10). Finding the most appropriate therapy for patients with relapsed/refractory disease in second and subsequent lines of therapy is far more complex and depends primarily on previously used agents and duration of response (8, 9).

Given the wide number of available treatment options for multiple myeloma, the optimal selection and sequencing of drugs is becoming progressively challenging (10–12). At the time of relapse, the choice of any subsequent line of therapy is driven by many patient- and disease-related factors, one of the most relevant being prior exposure, and eventually refractoriness, to one or more classes of novel agents (13). In particular, the widespread use of continuous lenalidomide as part of first-line therapies for both ASCT-eligible and -non eligible patients has progressively increased the population of patients who are lenalidomide-refractory in their second line of therapy (11, 14). Similarly, refractoriness to daratumumab and double refractoriness to both lenalidomide and daratumumab are on the increase, but reliable quantitative data are lacking (11, 14–16). Only recently have multiple studies and international guidelines begun to recommend the most appropriate and evidence-based treatment strategies for double- and triple-class exposed or refractory patients, who continue to face poor clinical outcomes with currently available options (10, 11, 17, 18). Breakthrough therapies with new mechanisms of action, including chimeric antigen receptor (CAR) T-cells and bispecific antibodies which directly harness immune effector cells to target the B-cell maturation antigen (BCMA) and other antigens, and even before the first-in-class BCMA-targeting drug conjugate belantamab-mafodotin, have demonstrated impressive efficacy in heavily pretreated patients in later treatment lines (12). Following the approval by the European Medicines Agency (EMA) of these breakthrough therapies for the treatment of triple-class exposed relapsed/refractory multiple myeloma, more recently ciltacabtagene autoleucel and belantamab-mafodotin in combination with standard-of-care agents have also been approved for the treatment of relapsed/refractory multiple myeloma after at least one prior therapy and are expected to be then reimbursed in Italy. Specifically, belantamab-mafodotin has a multimodal mechanism of action and is indicated for the treatment of relapsed or refractory multiple myeloma in combination with bortezomib and dexamethasone in patients who have received at least one prior therapy, and combined with pomalidomide and dexamethasone in patients who have received at least one prior therapy including lenalidomide (19). Ciltacabtagene autoleucel is indicated for the treatment of adult patients with relapsed and refractory multiple myeloma who have received at least one prior therapy, including an immunomodulatory agent and a proteasome inhibitor, and are refractory to lenalidomide (20).

The impact of newly approved therapies on clinical practice and the treatment algorithm for multiple myeloma in Italy remain uncertain. To explore these issues and address key areas of ambiguity in the current treatment landscape, we conducted a Delphi study designed to gather expert consensus on a series of statements concerning the epidemiology of multiple myeloma in Italy, current treatment patterns and unmet needs in later lines of therapy. Given the limited availability of robust empirical evidence in some of these areas, the Delphi method – an iterative, structured process for systematically collecting and refining expert opinion – was employed to support consensus-building in a rigorous and transparent manner (21).

## 2 Methods

Consensus on the topics of interest was reached using a modified Delphi procedure (21, 22). In this procedure, statements are submitted to a panel of experts who are asked to express their agreement or disagreement on each statement. Statements on which no consensus is reached are modified, if necessary, and resubmitted to the review by the expert panel until a predefined level of consensus is reached. The experts express their agreement or disagreement independently and anonymously. In each voting round, they have the opportunity to revise their responses after reviewing the aggregated results from the previous round. In the modified Delphi, the experts interact only at the final consensus meeting, where responses and statements with no consensus are discussed face-to-face.

### 2.1 Panel composition

For the modified Delphi process, hematology experts coming from 8 regions of Italy (Emilia-Romagna, Lazio, Marche, Piemonte, Puglia, Sicilia, Toscana, and Veneto) were involved. One expert served as chair key opinion leader of the study, whose main role was to ensure the appropriateness of the structure and contents of the statements and to interpret the results of the Delphi voting rounds and the final consensus meeting, and 8 experts constituted the expert panel; they participated in two rounds of voting and attended the final, virtual consensus meeting. The chair key opinion leader never interacted throughout the completion of the project with the expert panel and did not participate in the consensus meeting. Participants were selected based on a proven track record of relevant publications in the field of interest for this study, as well as proven clinical experience in managing patients with multiple myeloma in Italy. Furthermore, the majority of clinicians were already acquainted with the Delphi method, having previously participated in studies employing this approach.

IQVIA was responsible for drafting the statements, collecting and analyzing the results of the Delphi voting rounds and moderating the final consensus meeting. All participants were contacted individually via e-mail to confirm their interest in participating and throughout the Delphi process for technical support. Additionally, the chair key opinion leader participated in three virtual meetings to validate the statements and the results of the Delphi voting rounds.

### 2.2 Questionnaire design and structure

The areas of interest were identified based on a targeted review of the recent literature, clinical experience collected via a preliminary open survey on selected areas of interest, and the analysis of data from the IQVIA proprietary database Multiple Myeloma Integrated Patient Tracking. This database has been collecting quarterly data since the third quarter of 2018 on multiple myeloma patients in Italy and records information from a panel of 150 collaborating structures out of 210 centers treating multiple myeloma in Italy (estimated through a yearly pseudo-census survey sampling), covering more than 70% of treated Italian multiple myeloma patients and more than 75% of onco-hematology specialists. The open survey consisted of a preliminary version of the statements, along with a series of open-ended questions aimed at anonymously collecting numerical estimates from the clinicians on topics for which limited, or no published evidence was available. This preliminary phase served both to inform the drafting of the final version of the statements and to identify potential issues with their wording and structure prior to the first Delphi round. The mean values of the numerical estimates provided by clinicians were incorporated into the final version of statements 9, 14 and 18-19.

The final version of the questionnaire was drafted in Italian (the English translation of the questionnaire is included in **Supplementary Tables S1–S3`**) and was divided into three sections, corresponding to the areas of interest identified: 1) section I aimed at obtaining a consensus on epidemiology of multiple myeloma in Italy, including the validation of incidence and prevalence estimates, numbers of patients in the different lines of therapy, and evolution in the number of patients receiving second and subsequent lines of therapy in 2026-2028 [statements 1-11, **Supplementary Table S1**] 2) section II focused on the management of multiple myeloma and treatment patterns in clinical practice in Italy, including definition of most appropriate first-line therapies for ASCT candidates and non-ASCT candidates among those reimbursed in Italy at the time of statements drafting, rates of patients ineligible for lenalidomide, or refractory to both lenalidomide and daratumumab before second-line treatment, and second-line therapies for ASCT and non-ASCT candidates ineligible for lenalidomide, or refractory to both lenalidomide and daratumumab, among those reimbursed in Italy at the time of statements drafting [statements 12-21, **Supplementary Table S2**] 3) section III addressed the issue of current unmet needs in multiple myeloma management in second and subsequent lines of therapy and of clinicians’ perspectives on the expected unmet needs in patients with relapsed/refractory multiple myeloma in 2026-2028 (statements 22-31, **Supplementary Table S3**).

### 2.3 Delphi process

Between January and February 2025, IQVIA drafted a series of statements concerning the three areas of interest (sections I-III). The statements were reviewed by the chair key opinion leader, who provided feedback and suggestions for the refinement and finalization of the statements. Following digitalization of the questionnaire, a dedicated link to access the online platform used in the study was sent via e-mail to the 8 expert panel members, alongside an explanation of the objectives of the study and instructions on how to access the platform. The web-based questionnaire included an introductory paragraph explaining the objectives of each section, the characteristics of the questions included as well as the pre-determined definition of consensus. Panel experts were asked to express their agreement or disagreement on each statement using a 5-point Likert scale (1, Strongly disagree; 2, Disagree; 3, Neither agree nor disagree; 4, Agree; 5, Strongly agree) and were allowed to include relevant comments and notes under each statement. Consensus on “disagreement” or “agreement” was reached when ≥ 70% of the votes on each statement had a score in the ranges 1–2 or 4-5, respectively. Two rounds of voting were performed between February and March 2025. After the first voting round, anonymous responses were collected and aggregated to show the percentage of participants who selected each alternative. If deemed appropriate on the basis of collected responses and comments, statements could be modified to improve their clarity before submitting them to the second voting round. During the second round, only the statements that had failed to reach consensus of agreement or disagreement in the first round were submitted to the expert panel, to give the opportunity to participants to modify their answers if considered appropriate.

The final consensus meeting took place virtually in May 2025, was attended by 7 of the 8 expert panel members and was moderated by IQVIA. The meeting had the objective of presenting and discussing statements that did not reach consensus after the second round of voting and was followed by voting on each statement via a virtual poll system. The consensus definition for the virtual rounds was maintained for the consensus meeting, proportioned according to attendance.

## 3 Results

Most statements (28/31, 90%) achieved consensus in two rounds of Delphi voting, while 3 statements (statements 10, 22, 23) had to be further discussed and voted at the final meeting, where consensus was reached on all statements.

### 3.1 Epidemiology in Italy

These statements describe current epidemiology of multiple myeloma in Italy and the current and expected patient allocation to therapy lines (**Supplementary Table S1** - statements 1-11).

There was consensual agreement among the expert panel members about current estimates of the annual prevalence (approximately 34,000 individuals) and incidence (approximately 6,600 new cases in 2024) of multiple myeloma in Italy (3). The expert panel members also consensually agreed that in 2024, approximately 24,000 patients were managed at Italian centers, of whom 60% were treated with available therapeutic options, while the remaining were either in a watch-and-wait phase or were enrolled in clinical trials. The expert panel members also consensually agreed on the estimate that among treated patients, 56% were receiving first-line therapy, while 44% were undergoing treatment for relapsed/refractory disease. Of those receiving first-line therapy, 48% (4,000) were transplant-eligible, while 52% (4,300) were transplant-ineligible. The clinicians agreed that over the period 2021–2024 approximately 2,900 new patients per year initiated second-line therapy, and that in 2026–2028 this trend will decline slightly each year by approximately 2%. This estimate was based on the analysis of historical trends reported in the IQVIA database Multiple Myeloma Integrated Patient Tracking, and reflected clinicians’ perspectives that the increasing use of more effective first-line treatments in recent years will likely result in fewer patients requiring salvage second-line therapy over 2026-2028. Furthermore, the panel of experts agreed that, starting in 2026, approximately 35% of patients will receive second-line therapy due to disease progression post-transplant, while 65% will be transplant-ineligible and thus at higher risk of worse outcomes. From 2021 to 2024, approximately 2,100 new patients per year initiated third-line therapy, a figure expected to remain stable in 2026-2028. With regard to statement 10 that barely achieved consensus (71% agreement) at the third voting round, the panel members pointed to the difficulty of estimating the exact number of patients starting third-line therapy each year in Italy based on their caseload. However, they considered the source database sufficiently robust to support the reliability of the estimates. The 2024 data on the epidemiology and treatment of multiple myeloma in Italy validated via the Delphi process are summarized in **Table 1**, while **Table 2** summarizes the expected treatment prevalence patterns between 2026 and 2028.

**Table 1:** Multiple myeloma epidemiologic estimates and treatment prevalence in Italy as of 2024 validated using a modified Delphi process.

Summary of consensus	Estimate
Prevalent patients with multiple myeloma in Italy	~ 34,000
Incident patients with multiple myeloma in Italy	~ 6,600
Patients followed by Italian public centers treating multiple myeloma	~ 24,000
Patients treated with therapeutic options reimbursed in Italy for the treatment of multiple myeloma	~ 14,700 (61%)
undergoing 1L treatment	~ 8,300 (56%)
transplant-eligible	~ 4,000 (48%)
transplant-ineligible	~ 4,300 (52%)
undergoing 2L+ treatment	~ 6,400 (44%)

**Table 2:** Projected multiple myeloma epidemiologic estimates and treatment patterns in Italy between 2026 and 2028 validated using a modified Delphi process.

Summary of consensus	Estimate
Mean number of patients starting a 2L treatment each year between 2021 and 2024	~ 2,900
Projected increase/decrease in the number of patients receiving a 2L treatment between 2026 and 2028	- 2%
Expected proportion of transplant-eligible patients progressing to a 2L treatment between 2026 and 2028	35%
Expected proportion of transplant-ineligible patients progressing to a 2L treatment between 2026 and 2028	65%
Mean number of patients starting a 3L treatment each year between 2021 and 2024	~ 2,100
Projected increase/decrease in the number of patients receiving a 2L treatment between 2026 and 2028	0%

### 3.2 Treatment patterns in clinical practice in Italy

Consensus statements 12-21 summarized in **Supplementary Table S2** inform about the preferred physician’s choices of first- and second-line therapies in both transplant-eligible and transplant-ineligible patients and were formulated based on guidelines and treatment availability in Italy at the time of the Delphi process.

In line with published guidelines (9, 10), the expert panel members agreed that lenalidomide monotherapy is the preferred maintenance treatment in newly diagnosed transplant-eligible patients. For newly diagnosed, transplant-ineligible patients, the first-line treatment of choice is the triplet daratumumab-lenalidomide-dexamethasone. Clinicians confirmed that patients are considered refractory to lenalidomide if disease progression occurs during treatment or within two months after discontinuation (23). In nearly all patients refractory to lenalidomide or unable to tolerate it, a lenalidomide-based regimen is no longer a viable treatment option. As a consequence, in transplant-eligible patients, the reimbursed second-line therapies available in Italy are lenalidomide-free combinations, some of which include daratumumab. These therapies are listed in statement 16 and include: selinexor-bortezomib-dexamethasone, carfilzomib-dexamethasone, daratumumab-pomalidomide-dexamethasone, daratumumab-bortezomib-dexamethasone, isatuximab-carfilzomib-dexamethasone, and pomalidomide-bortezomib-dexamethasone. Among these, isatuximab-carfilzomib-dexamethasone was considered by the panelists as the most effective second-line therapy for patients without cardiovascular risk factors (statement 15). By contrast, transplant-ineligible patients who have received daratumumab-lenalidomide-dexamethasone as first-line therapy have less second-line options. In Italy, the reimbursed regimens include the following daratumumab- and lenalidomide-free regimens: pomalidomide-bortezomib-dexamethasone, selinexor-bortezomib-dexamethasone, and carfilzomib-dexamethasone (statements 20-21). Panelists agreed that most transplant-ineligible patients (90%) who progress after first-line therapy with daratumumab-lenalidomide-dexamethasone are likely to be refractory to both lenalidomide and daratumumab. A summary of these findings is provided in **Table 3**.

**Table 3:** Current treatment patterns in Italian clinical practice reported by the participants in the Delphi study.

Summary of consensus
Treatment patterns in 1L
Lenalidomide monotherapy is reported as the preferred maintenance treatment in newly diagnosed transplant-eligible patients.
For transplant-ineligible patients, the first-line treatment of choice is the triplet daratumumab-lenalidomide-dexamethasone.
Treatment patterns for transplant-eligible patients in 2L
Nearly all patients exposed to lenalidomide are either considered refractory or unable to tolerate it, and hence a lenalidomide-based regimen is no longer considered a viable treatment option.
Among lenalidomide-free regimens, isatuximab-carfilzomib-dexamethasone is considered to have the most satisfactory efficacy profile for patients without cardiovascular risk factors.
Other lenalidomide-free regiments considered as viable alternatives include selinexor-bortezomib-dexamethasone, carfilzomib-dexamethasone, daratumumab-pomalidomide-dexamethasone, daratumumab-bortezomib-dexamethasone, isatuximab-carfilzomib-dexamethasone, and pomalidomide-bortezomib-dexamethasone.
Treatment patterns for transplant-ineligible patients in 2L
Available options in transplant-ineligible patients who are refractory to lenalidomide are limited to daratumumab- and lenalidomide-free regimens: pomalidomide-bortezomib-dexamethasone, selinexor-bortezomib-dexamethasone, and carfilzomib-dexamethasone.
Most transplant-ineligible patients who progress after first-line therapy with daratumumab-lenalidomide-dexamethasone are considered refractory to both lenalidomide and daratumumab. In this population of patients, treatment options are limited to pomalidomide-bortezomib-dexamethasone, selinexor-bortezomib-dexamethasone, and carfilzomib-dexamethasone.

### 3.3 Unmet needs in the second and subsequent lines of therapy

These statements 22-31 highlight current unmet needs in the treatment of relapsed/refractory multiple myeloma in Italy (**Supplementary Table S3**). Despite the availability of multiple drugs and drug combinations, there continues to be a recognized unmet need for improved therapies which can overcome current limitations of available therapeutic options, with new mechanisms of action in both the second- and third-line setting.

Indeed, patients with multiple myeloma who progress after transplantation and are refractory or intolerant of lenalidomide face limited effective second-line treatments. In addition, for some of them additional cost of toxicities and logistical issues should be highlighted (statements 22-23). For example, isatuximab-carfilzomib-dexamethasone requires frequent access to the hospital for drug administration and exhibits unfavorable cardiovascular toxicities requiring intensive monitoring and careful patient selection. On the other hand, several alternative regimens which are not associated with cardiotoxic events, including selinexor-bortezomib-dexamethasone, daratumumab-pomalidomide-dexamethasone, daratumumab-bortezomib-dexamethasone, and pomalidomide-bortezomib-dexamethasone, are likely to be less effective, according to the consensus reached among the clinicians (statements 24-26).

The therapeutic options currently available in Italy for transplant-ineligible patients who at the time of first and second relapse are refractory to lenalidomide or to both lenalidomide and anti-CD38 monoclonal antibodies are very limited and were consensually perceived by the expert panel members as unsatisfactory in terms of both efficacy and safety profile (statements 24-26, 30). The expected introduction into the therapeutic armamentarium of immunotherapies with novel mechanisms of action, such as belantamab-mafodotin combined with novel agents and ciltacabtagene autoleucel, may address these gaps and will provide effective treatment options in this setting (statement 31). An overview of the identified unmet needs of current treatment of refractory/relapsed multiple myeloma in Italy is provided in **Table 4**.

**Table 4:** Current unmet needs reported by the participants in the Delphi study and expected impact of upcoming novel treatments.

Summary of consensus
Reported unmet needs in transplant-eligible patients in 2L
Currently reimbursed treatment options for patients with multiple myeloma who progress after transplantation and are refractory to lenalidomide, or do not tolerate lenalidomide, are considered not satisfactory in terms of efficacy and safety.
In patients refractory to lenalidomide who progress after transplantation, isatuximab-carfilzomib-dexamethasone is perceived as the regimen with the greatest efficacy but as demanding in terms of patient management and safety. The other available regimens have demonstrated a more favorable safety profile but are considered less effective.
Reported unmet needs in transplant-ineligible patients in 2L
The reimbursed treatment options with a satisfactory efficacy profile for transplant-ineligible patients who progress and are refractory to lenalidomide, or for whom lenalidomide-based second-line regimens are unsuitable, are consensually perceived as limited.
In transplant-ineligible patients with disease progression after first-line therapy, who are refractory to lenalidomide and daratumumab, the therapeutic options currently reimbursed for second-line therapy are considered unsatisfactory in terms of efficacy and safety.
Reported unmet needs in patients in 3L
In patients requiring third-line therapy, currently reimbursed options have shown unsatisfactory outcomes in clinical practice.
Impact of novel treatments
There is a perceived unmet need for new agents demonstrating better efficacy and safety than those of currently available regimens and acting via a distinct mechanism of action. Upcoming treatment options with novel mechanisms of action for second and subsequent lines of therapy (e.g., belantamab-mafodotin, ciltacabtagene autoleucel) are expected to address current unmet clinical needs of patients refractory or previously exposed to lenalidomide and/or daratumumab.

## 4 Discussion and conclusions

This study presents a series of consensus statements outlining the current epidemiology of multiple myeloma in Italy, treatment patterns in clinical practice, unmet clinical needs, and the anticipated changes in the management of patients with multiple myeloma over the next three years, following the expected introduction in Italy of two new immunotherapies in 2026. Notably, it provides structured, expert-derived insights into critical elements of current clinical practice, as well as on future trends within the evolving therapeutic landscape where published evidence remains limited. The consensus achieved on nearly all statements, and within two Delphi rounds, reinforces the robustness and consistency of the expert panel’s perspectives on key aspects of multiple myeloma management.

The estimates concerning the prevalence and incidence of multiple myeloma validated by the expert panel and in line with evidence reported by other authors (24, 25) confirm the significant burden of this condition in Italy. Moreover, the Delphi process allowed to validate the current estimates of treatment prevalence in Italian centers, highlighting that most patients diagnosed with multiple myeloma are actively undergoing pharmacologic treatment across all lines of therapy.

Regarding current management, the study allowed to confirm that the current clinical practice is aligned on considering lenalidomide maintenance and the triplet daratumumab-lenalidomide-dexamethasone as the preferred first-line choices in post-transplant and non-transplant eligible patients, respectively. As lenalidomide is part of the backbone of current first-line therapies, most patients relapsing after ASCT are lenalidomide-refractory and are not deemed eligible for receiving lenalidomide-based regimens in the second-line setting. Among lenalidomide-free therapies available for these patients, the triplet isatuximab-carfilzomib-dexamethasone was considered one of the most active, though it is associated with a relevant burden on patients in terms of management. Therefore, after balancing efficacy and safety the expert panel expressed a sub-optimal level of satisfaction with currently available therapeutic options in Italy in this setting. Similarly, the results of our study reflect the perception that a growing number of patients (≥ 90%) who are non-transplant eligible and receive first-line therapy with daratumumab-lenalidomide-dexamethasone, at the time of relapse are double refractory to both lenalidomide and daratumumab. This observation is consistent with other published data (25). Provided that published guidelines recommend the early use of daratumumab-lenalidomide-dexamethasone in the first-line settings, it is likely that the population of double-refractory patients will face a growing unmet need as the effectiveness and safety of currently available treatment options will be insufficient in a large proportion of them (8–11). Indeed, none of the treatment options for these patients currently available in Italy (i.e., pomalidomide-bortezomib-dexamethasone, selinexor-bortezomib-dexamethasone, and carfilzomib-dexamethasone) were formally investigated in the double-refractory setting of relapsed/refractory multiple myeloma, and data supporting the possible efficacy of some of them in limited series of patients are simply anecdotal.

As for the expected evolution in the management of multiple myeloma in the near future in Italy, the number of patients who will receive second-line therapy in 2026–2028 is predicted to decrease slightly but continuously, mostly due to the efficacy of current first-line therapies, while the number of patients receiving third-line therapy will be stable. Considering the significant efficacy of available first-line options and the better prognosis of ASCT eligible patients, as well as the increased adoption of daratumumab-lenalidomide-dexamethasone for the treatment of patients ineligible for ASCT, according to the consensus reached among clinicians it can be expected that most patients progressing to second-line therapy in the near future will be frail and possibly characterized by a worse prognosis in second- and subsequent lines of therapy.

The need for treatments with new mechanisms of action that have been evaluated in difficult-to-treat populations (e.g., double-refractory patients) are widely acknowledged among clinicians. Therefore, the expert panel members in our study expressed great expectations about the ability of immunotherapies (e.g., belantamab mafodotin-pomalidomide-dexamethasone, belantamab mafodotin-bortezomib-dexamethasone, ciltacabtagene-autoleucel) to meet the needs of current second-line treatment of multiple myeloma. In addition, the expert panel members also agreed about the insufficient effectiveness and safety of current third-line therapies.

This study has some limitations. Notably, the limited size and homogeneity of the expert panel may have influenced the findings, resulting in opinions and predictions that do not fully reflect the heterogeneity of clinical practice across Italy. However, the inclusion of participants with a proven experience in managing multiple myeloma patients working in reference centers across Italy was considered appropriate to capture relevant opinions in current clinical practice. In addition, the initial survey involving the expert panel members may have influenced the responses in following rounds. This survey was nevertheless useful for collecting insights for which limited or no evidence was available at the time of statement drafting. The timeframe considered (three years from the introduction of the new treatment options) is relatively short and may not represent long-term trends relevant for the therapeutic area considered in the present study. However, given the rapid and ongoing evolution in the availability of new therapies and management recommendations for multiple myeloma, extending the timeframe might have compromised the reliability of the estimates. Finally, the findings of this study should be interpreted in light of the Italian healthcare context in which it was conducted, including country-specific epidemiological data and treatment patterns. Differences in national clinical guidelines, physician preferences, and availability of therapies may limit the extent to which these results can be generalized to other healthcare settings or countries.

In conclusion, there is clearly a continuous need for novel treatment options for patients with relapsed/refractory multiple myeloma, as the increasing use of effective combined regimens, including triplets and quadruplets, as frontline treatment of newly diagnosed patients is leading to growing numbers of patients exposed to multiple drug classes who eventually become refractory. The present Delphi study has enabled to capture the perceptions of expert clinicians about the current treatment algorithm of multiple myeloma and the anticipated implications of adopting novel therapies in the near future in Italy. More in detail, the study has highlighted clinicians’ views on the current and future unmet needs in multiple myeloma management and the potential of upcoming therapies to address these gaps. Treatment options, along with the dynamics of refractoriness, are evolving rapidly. To optimally implement novel treatments in clinical practice, reliable tools for predicting this evolution are needed.

## Data Availability

The original contributions presented in the study are included in the article/**Supplementary Material**. Further inquiries can be directed to the corresponding author.
