# Supplementary material for: Addressing unmet needs in relapsed/refractory multiple myeloma: an Italian Delphi consensus on current challenges and emerging therapies
Source: Front Oncol. 2026 Feb 13;16:1756247. doi: 10.3389/fonc.2026.1756247 (PMC12945771; doi:10.3389/fonc.2026.1756247)
Supplement: Supplementary file 1 [file Table1.docx]

**Supplementary Table S1.** Consensus statements about the epidemiology of multiple myeloma in Italy

The percentages represent the proportion of votes with scores ranging from 4 to 5 (agreement) obtained during the Delphi voting rounds. This applies to all statements except statement 7, for which consensus was reached on disagreement (≥ 70% of votes with scores 1-2).

| **Statement first round** | **Statement second round** | **First round** | **Second round** | **Consensus meeting** |
| --- | --- | --- | --- | --- |
| **1.** The 2024 AIOM-AIRTUM report estimates that there are approximately 34,000 individuals affected by multiple myeloma in Italy. This value reflects the true prevalence of the disease in Italy. | **1.** The 2024 AIOM-AIRTUM report estimates that there are approximately 34,000 individuals affected by multiple myeloma in Italy. This value reflects the true prevalence of the disease in Italy. | 50.0% | 75.0% | --- |
| **2.** According to the 2024 AIOM-AIRTUM report, the incidence of multiple myeloma in Italy is approximately 6,600 new cases per year. This value reflects the true incidence of the disease in Italy. |  | 75.0% | --- | --- |
| **3.** Of the total number of patients with multiple myeloma referring to Italian centers in Q2 2024 (approximately 24,000), approximately 14,700 (61%) were in treatment with currently available therapeutic options. The remaining portion were patients in the watch-and-wait phase and patients participating in clinical trials. |  | 75.0% | --- | --- |
| **4.** Of the total number of patients treated at Italian centers in Q2 2024, approximately 8,300 (56%) were receiving first-line therapy and approximately 6,400 (44%) had relapsed/refractory multiple myeloma and were receiving subsequent lines of therapy. |  | 75.0% | --- | --- |
| **5.** Of the total number of patients receiving first-line therapy at Italian centers in Q2 2024, approximately 4,000 (48%) were transplant-eligible and in the induction or maintenance phase, while approximately 4,300 (52%) were transplant-ineligible. |  | 87.5% | --- | --- |
| **6.** Over the past 3 years (Q3 2021-Q2 2024), in Italy on average approximately 2,900 new patients started second-line therapy each year. | **6.** Over the past 3 years (Q3 2021-Q2 2024), in Italy on average approximately 2,900 new patients started second-line therapy each year. | 50.0% | 75.0% | --- |
| **7.** Over the next 3 years (2026-2028), the number of new patients receiving second-line therapy is expected to remain stable (approximately 2,900). | **7.** Over the next 3 years (2026-2028), the number of new patients receiving second-line therapy is expected to remain stable (approximately 2,900). | 62.5% | 87.5% | --- |
| **8.** Over the next 3 years (2026-2028), the number of new patients receiving second-line therapy will decline slightly (approximately ‒2% each year). | **8.** Over the next 3 years (2026-2028), the number of new patients receiving second-line therapy will decline slightly (approximately ‒2% each year). | 62.5% | 87.5% | --- |
| **9.** Starting from 2026, each year approximately 35% of individuals receiving second-line therapy will be patients with post-transplant disease progression. |  | 87.5% | --- | --- |
| **10.** Over the past 3 years (Q3 2021-Q2 2024), each year approximately 2,100 new patients on average started third-line therapy in Italy. | **10.** Over the past 3 years (Q3 2021-Q2 2024), each year approximately 2,100 new patients on average started third-line therapy in Italy. | 50.0% | 62.5% | 71.4% |
| **11.** Over the next 3 years (2026-2028), the number of new patients receiving third-line therapy will remain stable. | **11.** Over the next 3 years (2026-2028), the number of new patients receiving third-line therapy will remain stable. | 62.5% | 75.0% | --- |

AIOM-AIRTUM, Associazione Italiana di Oncologia Medica-Associazione Italiana Registri Tumori; Q2 2024, second quarter of 2024.

**Supplementary Table S2.** Consensus statements about current treatment patterns of patients with multiple myeloma in second and subsequent lines of therapy in Italy

The percentages indicate the proportions of votes with scores in the range 4-5 (agreement) achieved in the Delphi voting rounds.

| **Statement first round** | **Statement second round** | **First round** | **Second round** | **Consensus meeting** |
| --- | --- | --- | --- | --- |
| **12.** In newly diagnosed patients undergoing ASCT, the treatment of choice for maintenance is lenalidomide monotherapy. |  | 87.5% | --- | --- |
| **13.** In clinical practice, patients are considered refractory to lenalidomide if they progress during treatment with lenalidomide-based regimens or within 2 months from the last lenalidomide dose. |  | 100% | --- | --- |
| **14.** Given 100 transplant-eligible patients who have experienced disease progression during maintenance therapy with lenalidomide, or have discontinued lenalidomide due to toxicity, a second-line lenalidomide-based regimen is not advisable in almost all of them (90%-95%). |  | 100% | --- | --- |
| **15.** In transplant-eligible patients who experience disease progression and for whom a subsequent lenalidomide-based regimen is not advisable, second-line therapies providing the most satisfactory efficacy outcomes in clinical practice include the combination isatuximab-carfilzomib-dexamethasone, provided that cardiovascular risk factors are lacking, or the combination pomalidomide-bortezomib-dexamethasone. | **15.** In transplant-eligible patients who experience disease progression and for whom a subsequent lenalidomide-based regimen is not advisable, the second-line therapy providing the most satisfactory efficacy outcomes in clinical practice is the combination isatuximab-carfilzomib-dexamethasone, provided that cardiovascular risk factors are lacking. | 37.5% | 100% | --- |
| **16.** In Italy, the reimbursed second-line therapy options currently used in clinical practice for the treatment of transplant-eligible patients who experience disease progression, or for whom a new lenalidomide-based regimen is not advisable, include selinexor-bortezomib-dexamethasone, carfilzomib-dexamethasone, daratumumab-pomalidomide-dexamethasone, daratumumab-bortezomib-dexamethasone, isatuximab-carfilzomib-dexamethasone, and pomalidomide-bortezomib-dexamethasone. |  | 100% | --- | --- |
| **17.** In newly diagnosed, transplant-ineligible patients, the first-line therapy of choice is the combination daratumumab-lenalidomide-dexamethasone. |  | 87.5% | --- | --- |
| **18.** Given 100 transplant-ineligible patients who experience disease progression after first-line therapy with daratumumab-lenalidomide-dexamethasone, or have discontinued lenalidomide due to toxicity, a second-line lenalidomide-based regimen is not advisable in almost all of them (90%-95%). |  | 100% | --- | --- |
| **19.** Given 100 transplant-ineligible patients who experience disease progression after first-line therapy with daratumumab-lenalidomide-dexamethasone, approximately 90% will become refractory to both lenalidomide and daratumumab. |  | 100% | --- | --- |
| **20.** In transplant-ineligible patients who experience disease progression after first-line therapy with daratumumab-lenalidomide-dexamethasone and for whom a new lenalidomide-based regimen is not advisable, the second-line therapies of choice are limited to the combinations pomalidomide-bortezomib-dexamethasone and selinexor-bortezomib-dexamethasone. | **20.** In transplant-ineligible patients who experience disease progression after first-line therapy with daratumumab-lenalidomide-dexamethasone and for whom a new lenalidomide-based regimen is not advisable, the second-line therapies of choice are limited to pomalidomide-bortezomib-dexamethasone, selinexor-bortezomib-dexamethasone, and carfilzomib-dexamethasone. | 62.5% | 100% | --- |
| **21.** In transplant-ineligible patients who experience disease progression after first-line therapy with daratumumab-lenalidomide-dexamethasone and are refractory to both lenalidomide and daratumumab, second-line therapy options are limited to pomalidomide-bortezomib-dexamethasone, selinexor-bortezomib-dexamethasone, and carfilzomib-dexamethasone. |  | 100% | --- | --- |

ASCT, autologous stem cell transplant.

**Supplementary Table S3.** Consensus statements about unmet needs of multiple myeloma management in the second and subsequent lines of therapy

The percentages indicate the proportions of votes with scores in the range 4-5 (agreement) achieved in the Delphi voting rounds.

| **Statement first round** | **Statement second round** | **First round** | **Second round** | **Consensus meeting** |
| --- | --- | --- | --- | --- |
| **22.** In patients with post-transplant progression who are refractory to lenalidomide, or for whom it is not advisable to administer a lenalidomide-based regimen as second line, the currently available treatment options do not ensure satisfactory efficacy and safety. | **22.** In patients with post-transplant progression who are refractory to lenalidomide, or for whom it is not advisable to administer a lenalidomide-based regimen as second line, the currently available treatment options do not ensure satisfactory efficacy and safety. | 12.5% | 25.0% | 100% |
| **23.** In patients with post-transplant progression refractory to lenalidomide, or for whom a new lenalidomide-based regimen is not advisable, second-line therapy options with a satisfactory efficacy profile are limited to isatuximab-carfilzomib-dexamethasone. | **23.** In patients with post-transplant progression refractory to lenalidomide, or for whom a new lenalidomide-based regimen is not advisable, second-line therapy options with a satisfactory efficacy profile are limited to isatuximab-carfilzomib-dexamethasone. | 50.0% | 50.0% | 100% |
| **24.** In transplant-ineligible patients with disease progression after first-line therapy who are refractory to lenalidomide, or for whom a second-line lenalidomide-based regimen is not advisable, currently available treatment options do not ensure satisfactory efficacy and safety. |  | 25.0% | --- | --- |
|  | **25.** In transplant-ineligible patients with disease progression after first-line therapy who are refractory to lenalidomide, or for whom a second-line lenalidomide-based regimen is not advisable, therapeutic options with a satisfactory efficacy profile are limited. | --- | 100% | --- |
| **26.** In transplant-ineligible patients with disease progression after first-line therapy, who are refractory to lenalidomide and daratumumab, the therapeutic options currently available for second-line therapy do not ensure satisfactory efficacy and safety. | **26.** In transplant-ineligible patients with disease progression after first-line therapy, who are refractory to lenalidomide and daratumumab, the therapeutic options currently reimbursed for second-line therapy have provided unsatisfactory efficacy and safety results in clinical practice. | 50.0% | 75.0% | --- |
| **27.** There is a significant unmet clinical need for medications with demonstrated efficacy and safety in refractory patients, or in patients previously exposed to lenalidomide and/or daratumumab in the second-line setting. |  | 87.5% | --- | --- |
| **28.** There is a significant unmet need for medications with mechanisms of action distinct from those of the therapeutic options currently available in the second-line setting (monoclonal antibodies targeting CD28 and SLAMF7, immunomodulatory drugs, proteasome inhibitors, and selective inhibitors of nuclear export). |  | 100% | --- | --- |
| **29.** There is a significant unmet clinical need for medications with an improved safety profile in terms of frequency and severity of adverse events compared to the therapeutic options currently available for second-line therapy. | **29.** There is a significant unmet clinical need for medications with improved efficacy and safety compared to the therapeutic options currently available for second-line therapy. | 50.0% | 87.5% | --- |
| **30.** In patients progressing to third-line therapy, the currently reimbursed therapeutic options in this setting do not ensure satisfactory efficacy and safety results. | **30.** In patients progressing to third-line therapy, the currently reimbursed therapeutic options in this setting have demonstrated unsatisfactory efficacy and safety in clinical practice. | 62.5% | 87.5% | --- |
| **31.** Medications with novel mechanisms of action for second and subsequent lines of therapy, which will be available starting from 2026 (e.g., belantamab-mafodotin, ciltacabtagene autoleucel), will allow to address currently unmet clinical needs of patients refractory, or previously exposed to lenalidomide and/or daratumumab. |  | 75.0% | --- | --- |
